# miR-205-5p inhibits homocysteine-induced pulmonary microvascular endothelium dysfunction by targeting FOXO1: miR-205-5p inhibits pulmonary microvascular endothelial dysfunction

**DOI:** 10.3724/abbs.2023127

**Published:** 2023-07-25

**Authors:** Xiaobo Huang, Zhen Li, Ling Zhang, Yali Yang, Yanjia Wang, Sirui Li, Guizhong Li, Huiping Feng, Xiaoling Yang

**Affiliations:** 1 NHC Key Laboratory of Metabolic Cardiovascular Diseases Research Ningxia Medical University Yinchuan 750004 China; 2 School of Basic Medical Sciences Ningxia Medical University Yinchuan 750004 China; 3 Department of Respiratory and Critical Care Medicine Second Affiliated Hospital of Ningxia Medical University (The First People′s Hospital of Yinchuan) Yinchuan 750001 China; 4 Department of Pathology People’s Hospital of Ningxia Hui Autonomous Region Yinchuan 750004 China; 5 Department of Pathology General Hospital of Ningxia Medical University Yinchuan 750004 China; 6 Ningxia Baoshihua Hospital Yinchuan 750001 China

**Keywords:** homocysteine, miR-205-5p, FOXO1, DNA methylation, pulmonary microvascular endothelial dysfunction

## Abstract

Homocysteine (Hcy) is a risk factor for multiple chronic diseases, and vascular endothelial cell injury has been regarded as the initiating step for this process. miRNAs are involved in Hcy-induced endothelial dysfunction, while the underlying mechanism and roles of miRNAs in pulmonary endothelial dysfunction induced by homocysteine are unknown. Here, we find that miR-205-5p alleviates pulmonary endothelial dysfunction by targeting FOXO1 in CBS ^+/‒^ mice to protect against Hcy-induced pulmonary endothelial dysfunction. Mechanistically, we show that Hcy can lead to DNA hypermethylation of the miR-205-5p promoter due to the increased binding of DNMT1 to its promoter, which contributes to reduction of miR-205-5p expression. In summary, miR-205-5p promoter hypermethylation causes downregulation of miR-205-5p expression, resulting in a reduction in miR-205-5p binding to FOXO1 during homocysteine-induced pulmonary endothelial dysfunction. Our data indicate that miR-205-5p may be a potential therapeutic target against Hcy-induced pulmonary injury.

## Introduction

Homocysteine (Hcy) is a sulfur-containing amino acid and an intermediate metabolite in the body’s methionine cycle. Hyperhomocysteinaemia (HHcy) occurs when serum Hcy level reaches over 15 μM [1]. HHcy is one of the most important risk factors for cardiovascular [2], cerebrovascular [3] and peripheral vascular diseases [4]. A previous study showed that *ApoE*-knockout mice fed with a methionine-rich diet exhibited increased serum Hcy level and endothelial injury [5]. Glomerular podocytes were found to undergo apoptosis in an HHcy model replicated in cystathionine-beta-synthase ( *CBS*)-knockout mice, accompanied by renal dysfunction [6]. Therefore, Hcy may lead to vascular disease through injury to the endothelium. The increased production of oxygen free radicals and the activation of oxidative stress are important mechanisms for the abnormal morphology and function of the endothelium. The pulmonary endothelium is an important component of the alveolar-capillary membrane and plays an important role in gas exchange and in regulating the flow of liquids and soluble substances between the blood and the pulmonary interstitium. When pulmonary microvascular endothelial cells (PMVECs) are damaged or dysfunctional for a variety of reasons, this can lead to increased intercellular permeability [7] and, in severe cases, pulmonary edema [8]. However, it is not clear whether Hcy can damage PMVECs.

miRNAs are small, noncoding RNAs of approximately 18‒25 nt long that can affect disease progression by targeting and regulating the expressions of related genes. miRNAs regulate a variety of cellular functions, such as cell growth, differentiation, development, and apoptosis. miRNA dysregulation plays a key role in the pathophysiology of many diseases, such as cardiovascular disease, diabetes, neurodevelopmental disease, inflammatory disease, and cancer [9]. miRNA-205-5p (miR-205-5p) is a highly conserved miRNA that exists in many species and is abundant in epithelial cells. miR-205-5p can regulate epithelial cell function through many mechanisms and pathways [10]. In age-associated thymus involution, miR-205-5p inhibits thymic epithelial cell proliferation via FA2H-TFAP2A feedback regulation [11]. miR-205-5p can inhibit the proliferation, migration and tube formation of the human retinal endothelium under high glucose conditions, thus reducing high glucose-induced endothelial dysfunction. Inhibition of miR-205-5p increases the expressions of vascular endothelial growth factor-A and fibroblast growth factor-1, activates the extracellular signal-regulated kinase (ERK) signaling pathway, inhibits angiogenesis, and then promotes the formation of a malignant phenotype in gastric cancer [12]. Therefore, miR-205-5p may play an important role in the regulation of microvascular endothelial function. However, the mechanism of miR-205-5p in Hcy-induced PMVEC dysfunction is not clear.

The aim of this study was to examine the effect of Hcy on PMVEC dysfunction and to explore the role and mechanism of miR-205-5p in pulmonary endothelial dysfunction.

## Materials and Methods

### Animal models

Twelve SPF CBS ^+/‒^ male mice (C57BL/6 background; Jackson Laboratory, Bar Harbor, USA) weighing 18.0±2 g were selected and raised in the Experimental Animal Center of Ningxia Medical University and were randomly assigned into two groups: a model control group (fed with normal diet) and a high methionine group (fed with a high methionine diet). Meanwhile, six C57BL/6 mice were selected as the normal control group (fed with normal diet). After 16 weeks of routine feeding, the mice were anaesthetized with ether, the eyeballs were removed, and blood was collected from the inner canthus artery. Lung tissues were collected and frozen at ‒80°C for subsequent analyses. This study was approved by the Ethics Committee of Ningxia Medical University (Approval No. NYDWZX-2018-083).

### Measurement of serum Hcy level

The serum Hcy concentration was determined using the cyclic enzyme method with an automatic biochemical analyser (7080; Hitachi, Tokyo, Japan) according to the instructions provided with the instrument.

### Hematoxylin and eosin staining

The right lung tissue was fixed with 4% paraformaldehyde for 48 h, embedded in paraffin and cut into 4-μm-thick slices. Then, slices were incubated with hematoxylin for 5 min. After 10 s of color separation under acidic conditions and 10 s of color separation in ammonia solution, the sample was stained with eosin for 2 min, dehydrated with pure alcohol and cleared with xylene. Finally, the structure was observed with an inverted microscope (Olympus, Tokyo, Japan) using a neutral film. The pathology indexes were scored by evaluating alveolar congestion, intra-alveolar haemorrhage, lung oedema, interstitial infiltration of leukocytes and alveolar wall thickness. Semiquantitative analysis was performed according to each index based on the lesion range in each visual field at 200× magnification. Histopathological evaluation was analysed by three independent observers who were blinded to the experiment.

### Wet-to-dry weight ratio determination

To quantify the extent of pulmonary edema, the lung wet-to-dry weight ratios (W/D ratios) were calculated. In brief, the fresh weight of the upper lobe of the left lung was detected immediately after blotting the blood on filter paper. Then, the lung tissues were placed in a 65°C oven to dry for 48 h and reweighed again. Finally, the W/D ratio of lung tissue was calculated.

### Cell culture and grouping

Mouse PMVECs were purchased from BioWING Biotechnology (Shanghai, China) and cultured in RPMI-1640 medium containing 10% fetal bovine serum and 1% penicillin-streptomycin, in an incubator at 37°C with 5% CO _2_. All cell culture reagents were purchased from Gibco (Carlsbad, USA). When the cell fusion rate reached 80%, the cells were seeded into a 6-well plate at a density of 2×10 ^5^ cells/well. The cells were divided into a control group and an Hcy group. Then, the cells were collected after 24 h of treatment with or without 100 μM Hcy (Sigma-Aldrich, St Louis, USA) for subsequent experiments.

### Quantitative real-time polymerase chain reaction (qRT-PCR)

Total RNA was extracted from the cells using an RNA extraction kit (Thermo Scientific, Waltham, USA) according to the manufacturer’s instructions. The integrity of RNA was detected by agarose gel electrophoresis, and cDNA was synthesized using a cDNA reverse transcription kit (Thermo Fisher Scientific) according to the instructions of the manufacturer. Specific primers were designed using Primer 5.0 software (Premier Biosoft International, Palo Alto, USA). The primers for *FOXO1* and *miR-205-5p* were synthesized by Sangon Biotech (Shanghai, China), and the sequences are listed in Table 1. The amplification conditions were as follows: predenaturation at 95°C for 10 min, denaturation at 94°C for 30 s, annealing at 59°C for 30 s, extension at 72°C for 30 s, and amplification for 45 cycles. The relative changes of the target genes were analysed by the 2 ^‒ΔΔCT^ method. *U6* and *GAPDH* were used as controls.

**Table:** **Table1** Sequences of primers used for qRT-PCR

Gene	Forward primer (5′→3′)	Reverse primer (5′→3′)
*miR-205-5p*	CACGCCAGGCTCCA	CGGGCCCCCCGAACATT
*U6*	ATATATGGACGCTTCAATT	AACGCTTCGAATGCTTGT
*FOXO1*	AGTTCTTCTCTCTCTTGCACTCG	CTTCAAGAGAGAGAGAGAGCGCCAG
*DNMT3A*	GATGAGCCTGAGTATGAGGATGG	CAAGACACAATTCGGCCTGG
*DNMT3B*	CGTTAATGGGAACTTCAGTGACC	CTGCGTGTAATTCAGAAGGCT
*DNMT1*	CCGTGGCTACGAGGAGAAC	TTGGGTTTCCGTTTAGTGGGG
*GAPDH*	GGTTGCCCTGACTTCA	CCCTAGGCCCCCCCTGTTAT

### Western blot analysis

Cells were washed with cold PBS and centrifuged at 10,000 *g* for 5 min at 4°C after digestion with 0.25% trypsin. The total protein of each group was extracted using a protein extraction kit (KeyGene, Nanjing, China) and the protein concentration was quantified by the BCA method. Then, protein samples (20 μL) were separated by SDS-PAGE and transferred onto PVDF membranes. Then, 5% skimmed milk powder was used to block the membranes at 4°C overnight. The membranes were then incubated with primary antibodies against FOXO1 (1:1000; Abcam, Cambridge, USA), DNMT1 (1:1000; Abcam) or β-actin (1:2000; Abcam) at room temperature for 2 h. After incubation with horseradish peroxidase-conjugated (HRP) secondary antibody (1:10,000; Zhongshan Biotech, Guangzhou, China) for 1 h at room temperature, blots were then developed using enhanced chemiluminescence (ECL) solution. The protein bands were visualized on a GEL imaging system (Bio-Rad, Hercules, USA), and the protein levels were quantified by relative densitometry and normalized to that of β-actin as an internal control.

### Detection of MDA and SOD

The lung tissues and cells were mixed with cold saline (1:10; w/v), homogenized with a homogenizer, and then centrifuged at 3500 *g* for 10 min. The supernatant was collected and the levels of MDA and SOD in the supernatant were detected using commercial kits (Nanjing Jiancheng Bioengineering Institute, Nanjing, China) according to the respective instructions.

### Cell transfection

FOXO1 expression interference (si-FOXO1), miR-205-5p inhibitor, negative control inhibitor (NC inhibitor: 5′-CAGUACUUUUGUGUAGUA-3′), miR-205-5p mimic, and NC mimic (sense: 5′-UUCUCCGAACGUGUCACGUTT-3′, andantisense: 5′-ACGUGACACGUUCGGAGAATT-3′) were purchased from Gene Pharma (Shanghai, China). miR-205-5p mimic sequence is as follows: 5′-UCCUUCAUUCCACCGGAGUCUG-3′ and miR-205-5p inhibitor sequence is as follows: 5′-CAGACUCCGGUGGAAUGAAGGA-3. The siRNA sequences of FOXO1 are listed in Table 2. Recombinant adenoviruses expressing DNMT1 (Ad-DNMT1) were purchased from HANBIO (Shanghai, China). PMVECs (2×10 ^5^ cells/well) were seeded into six-well plates and transfected using Lipofectamine 2000 (Invitrogen, Carlsbad, USA) according to the manufacturer’s instructions.

**Table:** **Table 2** The siRNA sequences of *FOXO1*

Interference fragment	Sense (5′→3′)	Antisense (5′→3′)
si-NC	UUCUCCGAACGUGUCACGUTT	ACGUGACACGUUCGGAGAATT
si-FOXO1-2	CCCAGUCUGUCUGAAAUCATT	UGAUUUCAGACAGACUGGGTT
si-FOXO1-3	GCAACGAUGACUUUGAUAATT	UUAUCAAAGUCAUCGUUGCTT

### Double luciferase reporter experiments

The binding domain of miR-205-5p and the 3′ untranslated region (UTR) of *FOXO1* were obtained from the online database TargetScan. The 3′UTR of FOXO1 with wild-type and mutant binding sites for miR-205-5p was generated by YingBio Technology and cloned into the p-MIR-REPORT vector (YingBio Technology, Shanghai, China). The vectors were cotransfected with miR-205-5p mimic and mimic NC into PMVECs for 48 h, and then luciferase activities were measured and analysed using the dual-luciferase reporter assay system (Promega, Madison, USA) according to the manufacturer’s instructions.

### Nested methylation-specific polymerase chain reaction (nMS-PCR)

Genomic DNA was isolated from PMVECs using the TIANamp Genomic DNA Kit (Promega). The genomic sequence for the *miR-205-5p* gene and 2000 bases upstream was obtained from the UCSC genomic browser website ( www.urogene.org/cgi-bin/methprimer/methprimer.cgi). The primers for methylation analysis were designed based on this sequence by using MethPrimer. All primer sequences are listed in Table 3. The analysis was performed using quantitative real-time methylation-specific PCR. Amplification reactions were performed using 96-well plates. The thermocycling conditions were as follows: 95°C for 5 min, 50 cycles at 94°C for 10 s, 60°C for 45 s and 72°C for 1 min. The PCR products were subject to 2% agarose gel electrophoresis, and the optical density of methylated and nonmethylated bands was analysed with a Bio-Rad gel imaging system (Bio-Rad). The results were calculated as follows: methylation (%)=methylated OD value/(methylated OD value+nonmethylated OD value)×100%.

**Table:** **Table 3** Sequences of miR-205-5p primers for nMS-PCR

Gene	Species	Primer sequence (5′→3′)
Methylation primer	Mouse	Forward: GATAAATATTTCGTTGTTAGGGTTC
		Reverse: AATAAACTTCTTTTAAACTTACGCC
Unmethylation primer	Mouse	Forward: TAAATATTTTGTTGTTAGGGTTTGT
		Reverse: AATAAACTTCTTTTAAACTTACACC

### Phalloidin staining

F-actin was detected by using a phalloidin staining kit (Vazyme, Nanjing, China) according to the manufacturer’s instructions. In brief, cells were fixed with 4% paraformaldehyde for 15 min, and then a diluted phalloidin mixture was added and incubated at 37°C for 1 h. DAPI was added and incubated in the dark for 5 min, and F-actin was detected under a fluorescence microscope (Zeiss, Oberkochen, Germany).

### Statistical analysis

The experimental results were analysed by using GraphPad Prism 5.0 software (GraphPad Software, Inc., San Diego, USA). Data are presented as the mean±SD. Statistical comparisons among different groups were conducted by one-way ANOVA, while differences between two groups were assessed by Student’s *t* test. *P*<0.05 was defined as statistically significant.

## Results

### Hcy causes lung tissue structural changes

**Figure 1:**
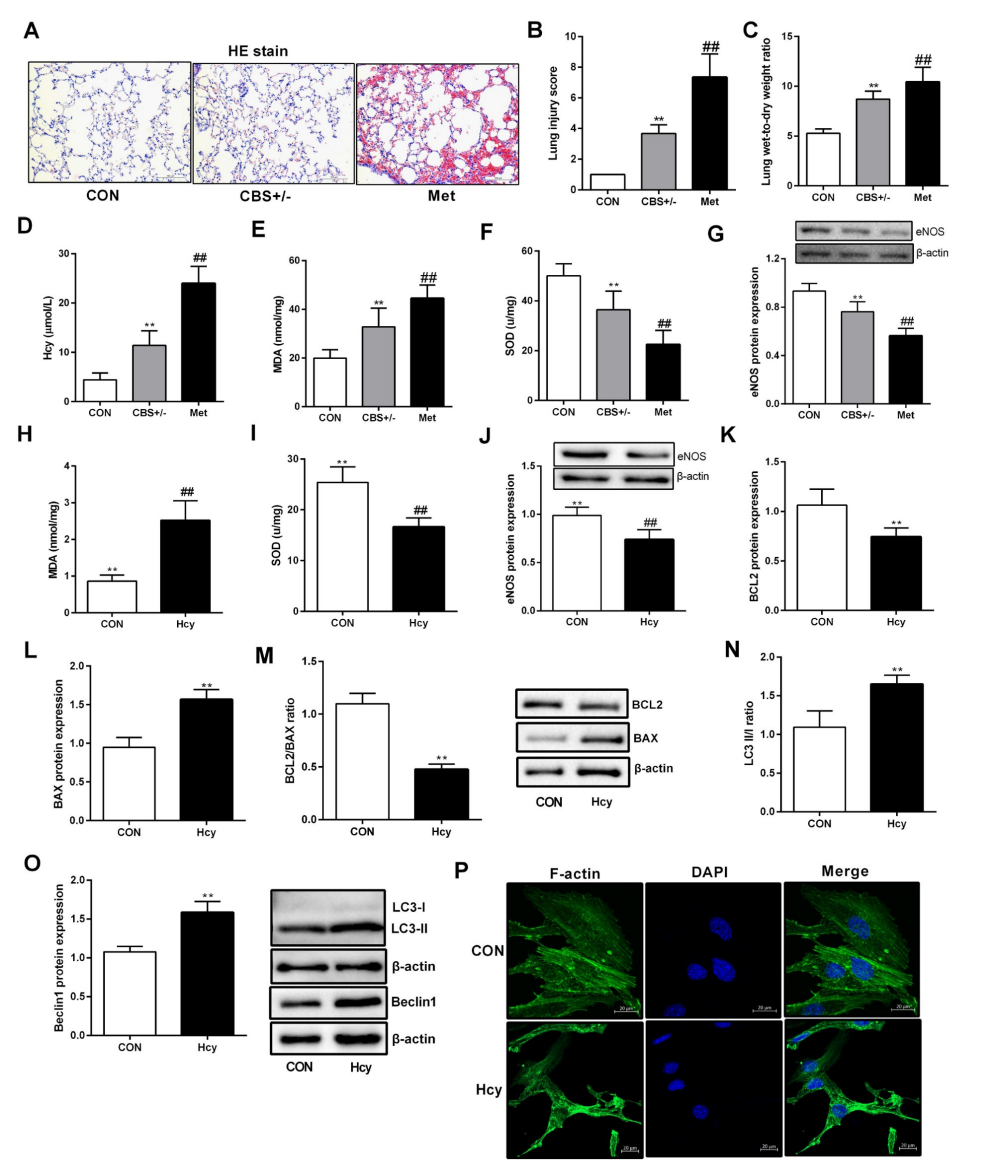
Figure 1 Effect of Hcy on the dysfunction of lung tissue and PMVECs (A) After 16 weeks of feeding, mice were sacrificed, and lung tissue was collected. Lung tissue structure changes were observed with HE staining in each group (20×). (B) Lung injury scores were calculated and compared among groups. (C) Lung wet-to dry weight ratio. (D) Serum Hcy level in mice was detected with an automatic biochemical analyser. (E,F) SOD and MDA levels in lung tissue were determined with a multifunctional microplate reader. (G) eNOS protein expression in lung tissues was measured by western blot analysis. (H,I) PMVECs were treated with 100 μM Hcy for 24 h, and SOD and MDA levels were detected. (J‒M) Total protein was extracted from PMVECs, and the protein expressions of eNOS, BCL2 and BAX were detected by western blot analysis. (N,O) The autophagy-related proteins LC3 and beclin1 were detected by western blot analysis after PMVECs were treated with Hcy. (P) F-actin expression in PMVECs was detected by phalloidin staining. Green fluorescence indicates F-actin, and the nuclei were stained with DAPI (blue). Scale bar: 20 μm. Data are presented as the mean±SD. ** P<0.01 vs CON group; ##P<0.01 vs CBS +/‒ group. CON, control.

In the body, Hcy is produced by methionine with trans-methylation [13]. One of the main pathways for Hcy is the synthesis of L-cysteine, which is catalyzed by cystathionine synthase (CBS) [14]. Blocking cystathionine-beta-synthase may lead to dysfunction of Hcy metabolism and an increase in Hcy level. As shown in Figure 1A, mice in the normal control group had normal lung tissue structure, while mice in the model control group (CBS ^+/‒^) exhibited excessive inflammatory cell infiltration and edematous alveolar walls. After CBS ^+/‒^ mice were fed with a high methionine diet, inflammatory infiltration was increased, and the damage to the pulmonary alveoli was more severe ( Figure 1B). The lung wet-to-dry weight ratio was also significantly increased in the high methionine group (Met) compared with that in the normal control group (CON) or the CBS ^+/‒^ group ( Figure 1C). Furthermore, the serum Hcy level in the Met group mice was significantly higher than that in the CBS ^+/‒^ group mice, indicating that the hyperhomocysteinaemia (HHcy) model was successfully replicated ( Figure 1D). In addition, the Met group mice also exhibited typical dysfunction characterized by decreased SOD and eNOS levels and increased MDA level ( Figure 1E‒G). After PMVECs were treated with Hcy, the trend of SOD, MDA and eNOS levels was consistent with that in the *in vivo* experiment ( Figure 1H‒J). Furthermore, the levels of apoptosis and autophagy in PMVECs were increased by Hcy ( Figure 1K‒O). The skeleton structure of F-actin was distributed unevenly and disorderly after treatment with Hcy, while it was distributed evenly in normal cells and did not accumulate or recombine to form stress fibres *in vitro* ( Figure 1P). These results indicated that Hcy could lead to lung structure injury and dysfunction both *in vivo* and *in vitro*.

### miR-205-5p mediates pulmonary endothelial dysfunction induced by Hcy

**Figure 2:**
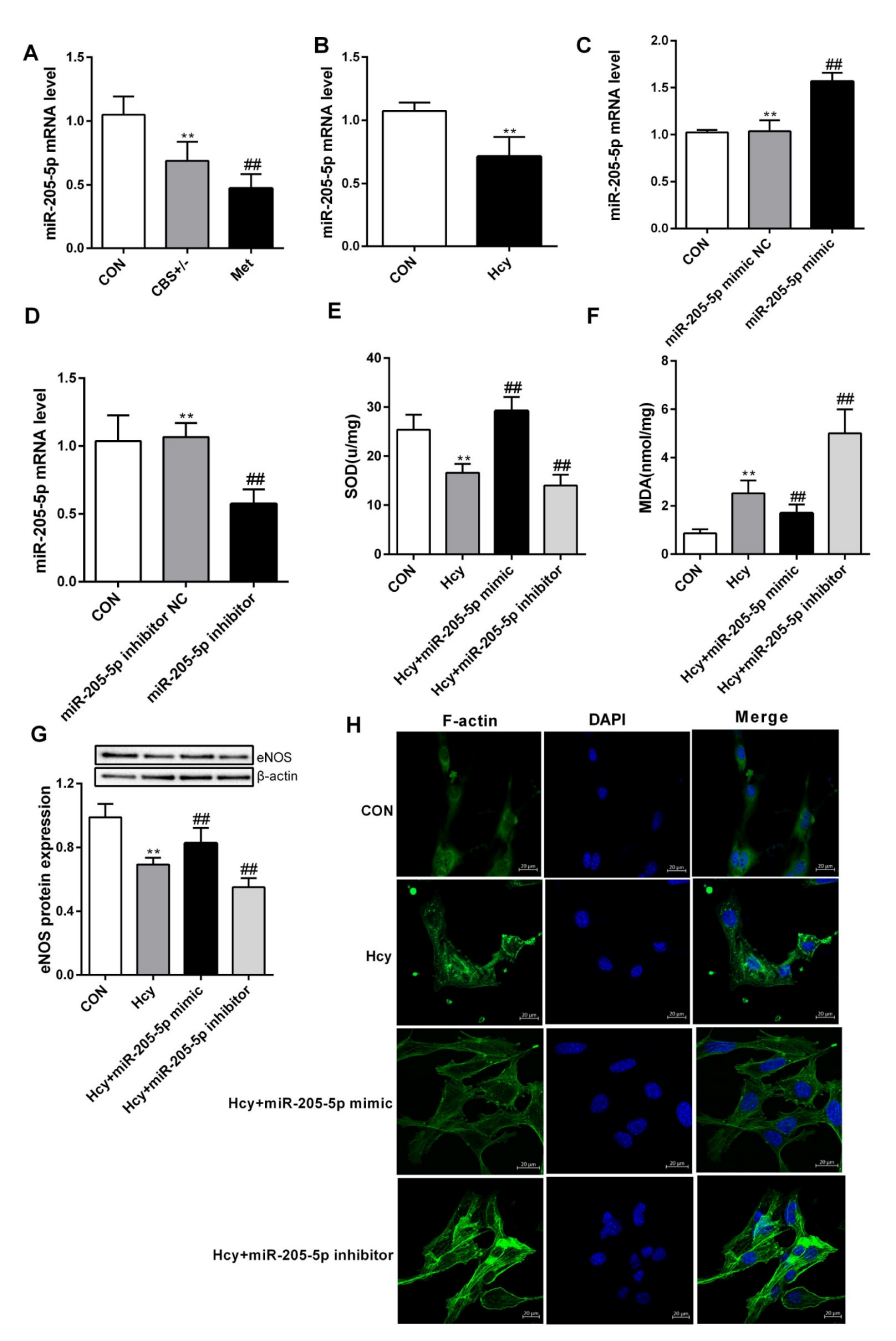
Figure 2 miR-205-5p mediates Hcy-induced pulmonary endothelial dysfunction (A,B) Total RNA was extracted from lung tissues and PMVECs and reverse transcribed into cDNA. Then, the mRNA level of miR-205-5p was measured by qRT-PCR. (C,D) miR-205-5p mRNA level was detected after transfection with miR-205-5p mimic or inhibitor, respectively. (E,F) SOD and MDA contents in cells were detected after transfection with miR-205-5p mimic or inhibitor. (G) The protein expression of eNOS was determined by western blot analysis after the cells were transfected with miR-205-5p mimic or inhibitor. (H) F-actin expression was detected by phalloidin staining in PMVECs after transfection with the miR-205-5p mimic or inhibitor. Green fluorescence indicates F-actin, and the nuclei were stained with DAPI (blue). Scale bar: 20 μm. Data are presented as the mean± SD. *P<0.05 vs CON group; ##P<0.01 vs CBS+/‒ group or Hcy group. CON, control.

miRNAs play important roles in the regulation of approximately 1/3 of human genes. It not only participates in the physiological processes of individual development, organ formation and material metabolism but also in the occurrence and development of various human diseases [9]. Under normal conditions, miR-205-5p may be expressed in lung tissue; however, qRT-PCR analysis showed that the expression level of miR-205-5p was downregulated in CBS ^+/‒^ mice, especially under conditions with a high methionine diet ( Figure 2A). Similar results were also obtained in PMVECs after Hcy treatment ( Figure 2B). To investigate the effect of miR-205-5p on lung endothelial function, we transfected a miR-205-5p mimic or inhibitor into PMVECs ( Figure 2C,D). As shown in Figure 2E‒H, miR-205-5p inhibitor transfection significantly decreased PMVEC function, while dysfunction of PMVECs induced by Hcy might be reversed at least partly by the miR-205-5p mimic, implying that Hcy accelerated PMVEC dysfunction via downregulation of miR-205-5p expression.

### miR-205-5p targets FOXO1

**Figure 3:**
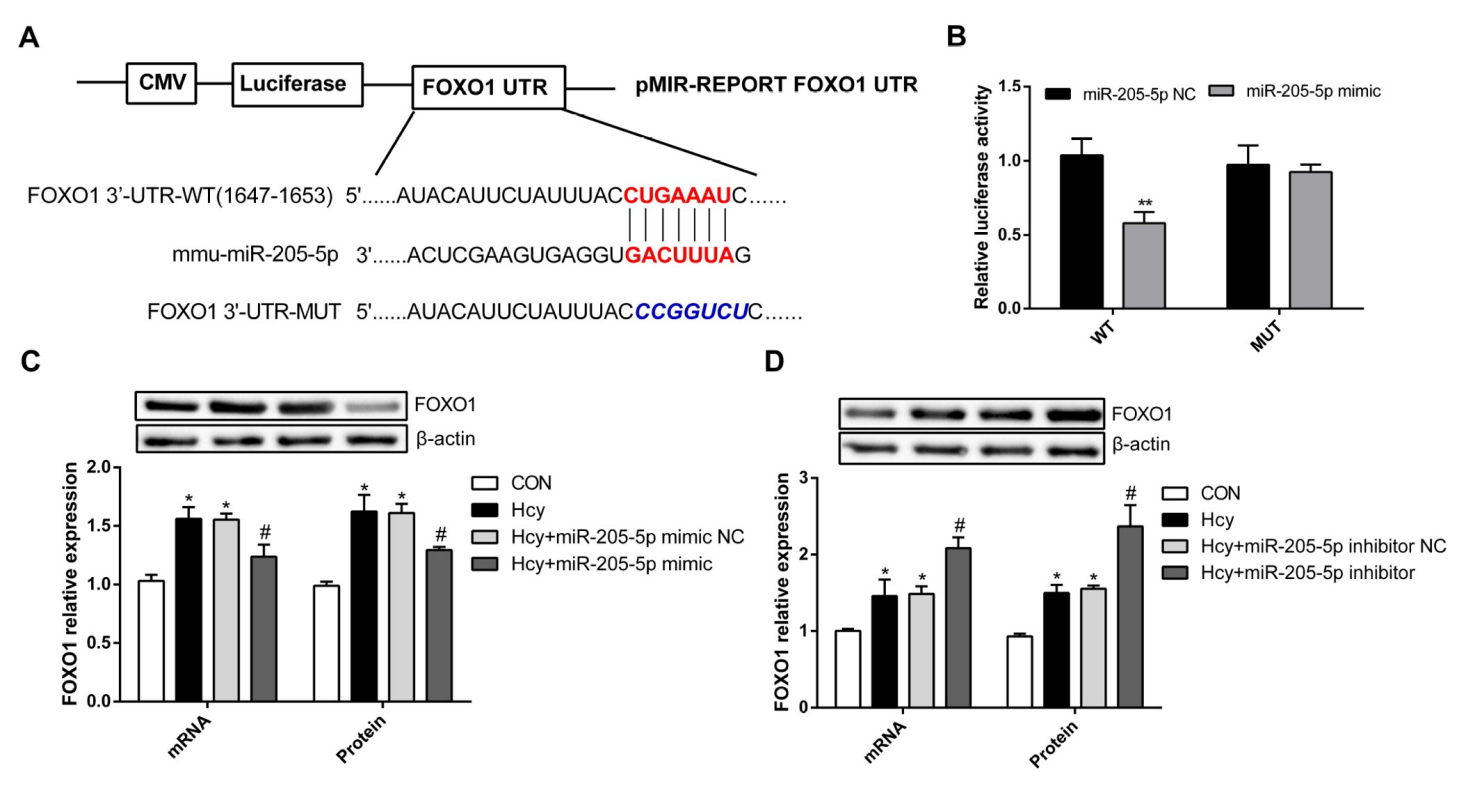
Figure 3 miR-205-5p inhibits FOXO1 expression (A) Bioinformatics predicted the targeted binding of the miR-205-5p seed sequence to the FOXO1 3′UTR. (B) A 3′UTR containing wild-type (WT) and mutant (MUT) FOXO1 genes was constructed and cotransfected with miR-205-5p mimic into PMVECs. The relative activity of luciferase is expressed by the ratio of the activity of firefly luciferase to that of algal luciferase. (C,D) The effect of the miR-205-5p mimic and inhibitor on FOXO1 mRNA and protein expression levels. Data are presented as the mean±SD. *P<0.05 vs CON group; #P<0.05 vs Hcy group. CON, control.

miRNAs play important roles in the development of disease because they regulate the transcription and translation of downstream target genes through the 3′UTR of the target gene. A previous study showed that miR-205-5p is a modulator of insulin sensitivity that inhibits FOXO function [15]. Moreover, in large and small follicles, miR-205 is highly expressed, and FOxO signaling pathways involve the oocyte meiosis pathway [16]. To identify the molecular mechanism by which miR-205-5p is involved in Hcy-induced PMVEC dysfunction, miR-205-5p and its potential target gene were predicted by TargetScan. Bioinformatics analysis showed that the seed sequence of miR-205-5p was associated with the 3′UTR of FOXO1 ( Figure 3A). In this study, wild-type (WT) and mutant (MUT) plasmids carrying the 3′UTR of *FOXO1* were constructed into vectors and cotransfected with the miR-205-5p mimic into PMVECs. The luciferase activity of the wild-type plasmid was significantly decreased ( Figure 3B); however, after *FOXO1* 3′UTR mutation, luciferase activity was not significantly changed. These results suggested that miR-205-5p could target *FOXO1* by binding to the putative sequences within its 3′UTR. Furthermore, overexpression of miR-205-5p significantly inhibited FOXO1 mRNA and protein expression levels, while FOXO1 expression was increased after downregulation of miR-205-5p expression ( Figure 3C,D). These results suggested that miR-205-5p could inhibit FOXO1 expression in Hcy-induced PMVEC dysfunction, indicating that miR-205-5p ameliorates Hcy-induced PMVEC dysfunction by targeting *FOXO1*.

### Effect of FOXO1 on PMVEC dysfunction induced by Hcy

**Figure 4:**
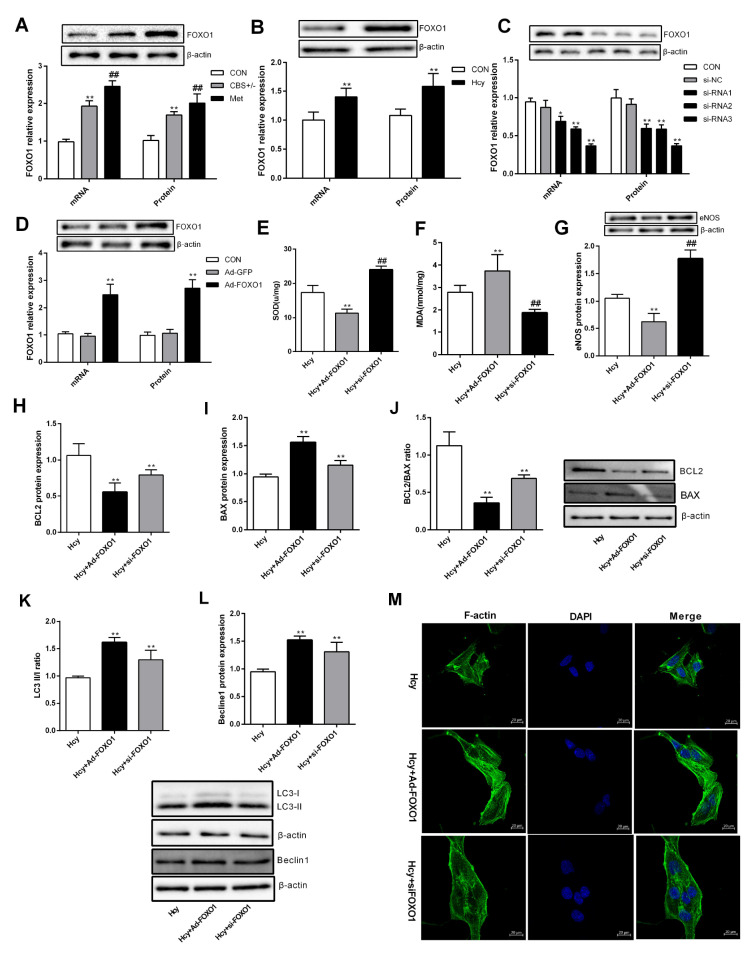
Figure 4 Effect of FOXO1 on PMVEC dysfunction induced by Hcy (A,B) Total RNA and protein were extracted from the lung tissue of mice and PMVECs, and the mRNA and protein expression levels of FOXO1 were measured by qRT-PCR and western blot analysis respectively. (C,D) The mRNA and protein expression levels of FOXO1 were measured by qRT-PCR and western blot analysis respectively after transfection of three FOXO1-siRNA fragments or Ad-FOXO1 into PMVECs. (E‒G) The contents of SOD and MDA and the protein expression of eNOS in PMVECs were detected after transfection with FOXO1-siRNA and recombinant adenovirus. (H‒J) BCL2 and BAX protein expressions were detected by western blot analysis, and the ratio of BCL2/BAX was calculated after FOXO1 overexpression or FOXO1 knockdown. (K,L) The autophagy-associated proteins LC3 and beclin1 were determined after PMVECs were transfected with Ad-FOXO1 or the interference fragment. (M) F-actin expression in PMVECs was detected by phalloidin staining after cells were transfected with si-FOXO1 or Ad-FOXO1. Green fluorescence indicates F-actin, and the nuclei were stained with DAPI (blue). Scale bar: 20 μm. Data are presented as the mean±SD. *P<0.05, ** P<0.01 vs CON group; #P<0.05, ## P<0.01 vs CBS+/‒ or Hcy group. CON, control.

FOXO1 is a transcription factor widely distributed in the heart, brain, lung and other tissues and organs [17]. FOXO1 plays physiological and pathophysiological roles through oxidative stress, autophagy and apoptosis [18]. In the present study, we focused on the role of FOXO1 in Hcy-induced dysfunction of PMVECs. Compared with that in the normal control group mice, the expression of FOXO1 in CBS ^+/‒^ mice was increased, especially in the high methionine group ( Figure 4A). After treatment with 100 μM Hcy, the expression of FOXO1 in PMVECs was also increased significantly ( Figure 4B). To clarify the role of FOXO1 in Hcy-induced pulmonary microvascular dysfunction, a synthetic FOXO1 interfering fragment and adenovirus carrying FOXO1 were further transfected into PMVECs ( Figure 4C,D). Interference with FOXO1 expression reversed PMVEC dysfunction characterized by SOD ( Figure 4E), MDA ( Figure 4F), eNOS ( Figure 4G), BCL2 and BAX ( Figure 4H‒J), LC3II/I and beclin1 ( Figure 4K,L), and F-actin ( Figure 4M), which suggested that FOXO1 might promote Hcy-induced PMVEC dysfunction.

### DNA methylation regulates miR-205-5p expression

**Figure 5:**
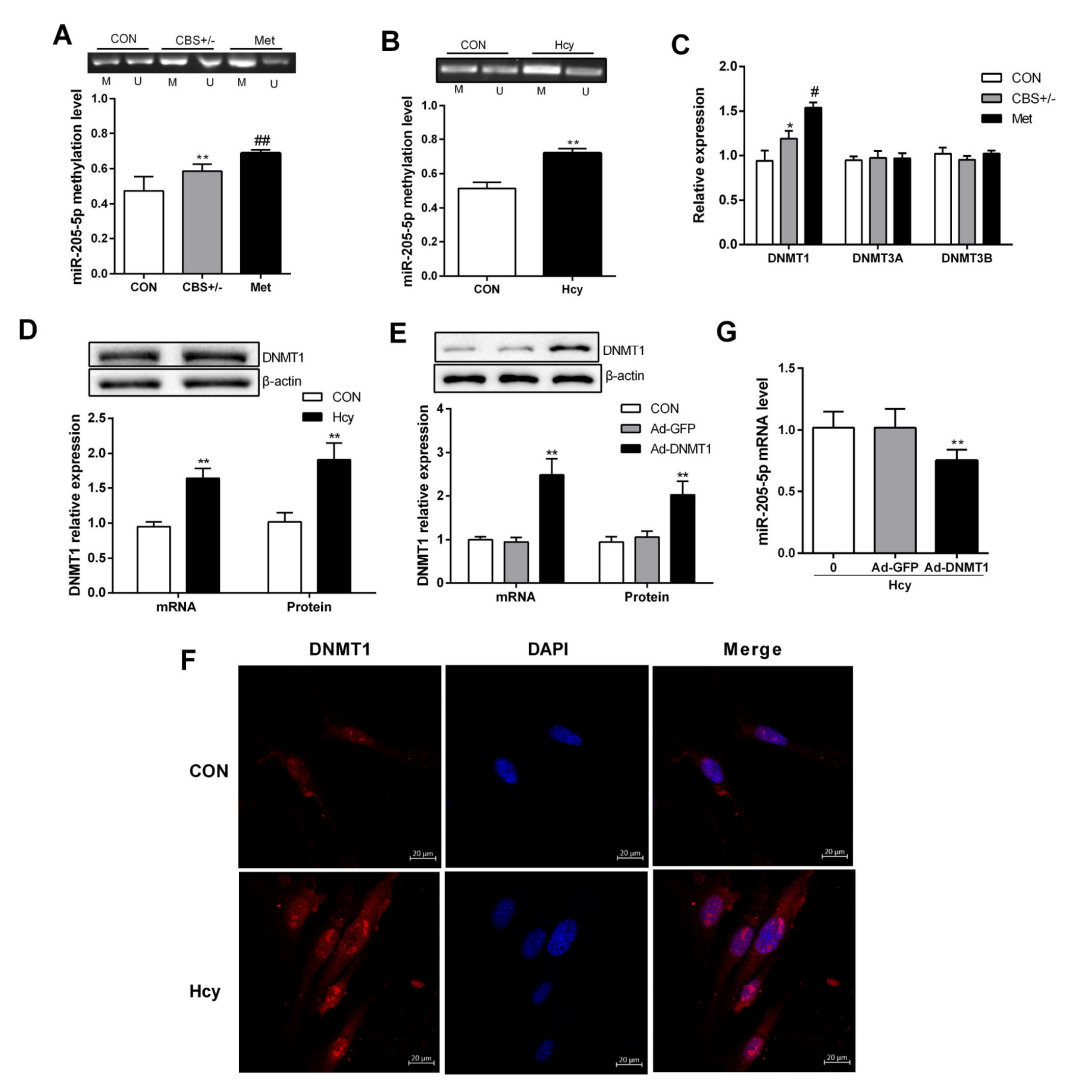
Figure 5 Hcy regulates miR-205-5p expression by affecting promoter DNA methylation (A,B) Genomic DNA of lung tissue and PMVECs was extracted and treated with sodium bisulfite, and the methylation level of the miR-205-5p promoter was detected by MS-PCR. (C) The mRNA levels of DNMT1, DNMT3A and DNMT3B were measured by qRT-PCR. (D) The mRNA and protein expression levels of DNMT1 in PMVECs were measured by qRT-PCR and western blot analysis, respectively. (E) The expression of DNMT1 was detected by quantitative PCR and western blot analysis after PMVECs were transfected with the DNMT1 overexpression plasmid. (F) Representative immunofluorescence images of PMVECs stained with DNMT1 (red) and DAPI (nuclear staining, blue). Scale bar: 20 μm. (G) The DNMT1 overexpression plasmid was transfected into PMVECs, and the expression of miR-205-5p was measured by qRT-PCR. Data are presented as the mean±SD. *P<0.05, **P<0.01 vs CON group; #P<0.05 vs CBS+/‒ group. CON, control.

DNA methylation is one of the earliest epigenetic modifications. It transfers the methyl group (CH3) to the CG base of the cytosine carbon atom by a covalent bond and usually occurs in the promoter region of a gene [19]. In general, DNA methylation can shut down the activity of certain genes, while demethylation induces gene reactivation and expression [20]. As shown in Figure 5A, the promoter of miR-205-5p was hypermethylated in CBS ^+/‒^ mice, which was augmented by a high methionine diet. The results of *in vitro* experiments were consistent with those of the animal experiments ( Figure 5B), suggesting that Hcy significantly increased DNA methylation of the miR-205-5p promoter.

In the body, DNA methylation is achieved through DNA methyltransferase. There are two classes of DNA methylases: maintenance DNA methyltransferase, DNMT1, and de novo methylases, including DNMT3A and DNMT3B [ 21, 22]. To better illustrate which DNMTs (DNMT1, DNMT3A, and DNMT3B) are involved in DNA methylation of the miR-205-5p promoter, the expressions of DNMTs in the lung tissue of CBS ^+/‒^ mice and PMVECs were determined ( Figure 5C,D). It was found that the expression of DNMT1 was significantly increased both *in vivo* and *in vitro*, indicating that DNMT1 contributes to hypermethylation of the miR-205-5p promoter. To clarify whether DNA methylation regulates the expression of miR-205-5p, we constructed a DNMT1 overexpression plasmid and transfected it into PMVECs ( Figure 5E,F).After DNMT1 overexpression, miR-205-5p expression was significantly decreased ( Figure 5F), suggesting that DNMT1 may be a key enzyme in the downregulation of miR-205-5p expression induced by Hcy.

## Discussion

Hcy is a sulfhydryl amino acid. *In vivo*, approximately 50% of Hcy can be remethylated to methionine by vitamin B _12_-dependent methionine synthetase, and the other 50% can be converted into L-cysteine and adenosine by vitamin B _6_-dependent CBS [ 5, 6]. Defects in or reduced activity of CBS can lead to increased blood Hcy level [6]. In this study, high methionine diet caused HHcy in CBS ^+/‒^ mice. The mechanism of Hcy-induced disease is related to the damage in the vascular endothelium. When Hcy enters the plasma, its own oxidation occurs very quickly. Then, Hcy disulfide, Hcy sulfur emulsion, *etc*., were produced, accompanied by a large amount of superoxide anion (O _2_^‒^), hydrogen peroxide (H _2_O _2_) and malondialdehyde via its own oxidation process [23]. When OFR production is increased, endothelial function is impaired, further leading to a decrease in nitric oxide (NO) and eNOS [24]. Pulmonary microendothelium is an important component of the alveolar-capillary membrane. PMVEC dysfunction may reduce alveolar gas exchange and, in severe cases, cause respiratory failure [8]. In this study, the lung tissue structure and function of mice fed with high methionine diet were disordered, and there was substantial exudation in the alveolar cavity, which indicated that high Hcy level could cause lung tissue structure disorder. At the same time, MDA was increased, while SOD and eNOS expressions were decreased in both high methionine-fed mice and Hcy-treated PMVECs. These results indicated that Hcy might cause structural and functional disorders in PMVECs.

miRNAs are a highly conserved family of noncoding RNAs (ncRNAs) with a length of ~22 nt. In animals, a single miRNA can recognize multiple targets, and a single mRNA target can be recognized by multiple miRNAs [9]. Based on the analysis of the 5′-end “seed” sequence homology of miRNAs, approximately two-thirds of the protein-coding genes in the human genome are regulated by miRNAs [21]. Because miRNAs have multiple target genes, their effects on different tissues and organs are also different. miR-205-5p is a highly conserved miRNA. Knockdown of *Malat1* alleviates high glucose-induced angiogenesis by regulating the miR-205-5p/VEGF-A axis [25]. miR-205-5p decreases angiogenesis by inhibiting *VEGFA* expression in extracellular vesicles isolated from diabetic foot ulcer wound fluid [26]. High expression of miR-205-5p *in vitro* increases the number of endothelial progenitor cells, reduces cell volume, and promotes cell growth [27]. Thus, miR-205-5p can influence the onset and development of disease by regulating endothelial function. Our results showed that the expression of miR-205-5p was decreased in both high methionine-fed mice and Hcy-treated PMVECs. Moreover, increased expression of miR-205-5p resulted in a decrease in MDA level and an increase of SOD and eNOS levels, suggesting that miR-205-5p might partly reverse the dysfunction of PMVECs induced by Hcy.

FOXO1 is an important member of the FOX family. FOXO1 is widely expressed in many organs and tissues, such as the myocardium [28], vascular endothelium [29] and liver [30]. FOXO1 is involved in many physiological and pathological processes, including cell proliferation, apoptosis and metabolism [31]. A previous study showed that FOXO1 expression is increased under oxidative stress, inflammation and other pathological conditions [32]. The decreased expression of FOXO1 in endometrial epithelial cells could lead to the differentiation, endocytosis and apoptosis of endometrial epithelial cells in the peri-implantation period. Myeloid-specific *FOXO1*-knockout mice can attenuate oxidative stress-induced hepatocyte damage [33]. Therefore, FOXO1 plays an important role in endothelial cell dysfunction and oxidative stress. Here, we found that the expression of FOXO1 was significantly increased both in the lung tissue of high methionine-fed mice and in Hcy-treated PMVECs. Interference with FOXO1 expression was able to reverse the endothelial dysfunction of PMVECs caused by Hcy, suggesting that Hcy might exert its damaging effect on the vascular endothelium by upregulating FOXO1.

As a transcription factor, FOXO1 is regulated by many factors, such as DNA methylation, histone modification, and miRNA regulation [31]. Through bioinformatics prediction, FOXO1 and miR-205-5p were found to have binding sites. Double luciferase reporter gene results verified that miR-205-5p can bind to the FOXO1 3′UTR and reduce FOXO1 expression, indicating that miR-205-5p could downregulate FOXO1 expression in Hcy-treated PMVECs. These results indicated that Hcy causes PMVEC dysfunction by downregulating the expression of miR-205-5p and subsequently increasing FOXO1 expression.

Since changes in miR-205-5p expression are associated with PMVEC dysfunction, it is necessary to understand the regulatory mechanism of miR-205-5p. One of the regulatory mechanisms for miR-205-5p is promoter methylation. In mammary epithelial cells, overexpression of ERBB2 can promote *miR-205-5p* methylation through Ras/Raf/Eek/Erk pathway-mediated DNMT expression [34]. Mel-18 prevents the recruitment of DNMT to the promoter of *miR-205-5p*, reducing the degree of methylation in its promoter region and then promoting the expression of miR-205-5p [35]. In this study, methylation of the *miR-205-5p* promoter was increased in high methionine-fed mice and in Hcy-treated PMVECs. Overexpression of DNMT1 decreased miR-205-5p expression and PMVEC dysfunction, indicating that hypermethylation of the *miR-205-5p* promoter is an important pathogenic mechanism during Hcy-induced PMVEC dysfunction and that DNMT1 is the key enzyme regulating the hypermethylation of miR-205-5p.

**Figure 6:**
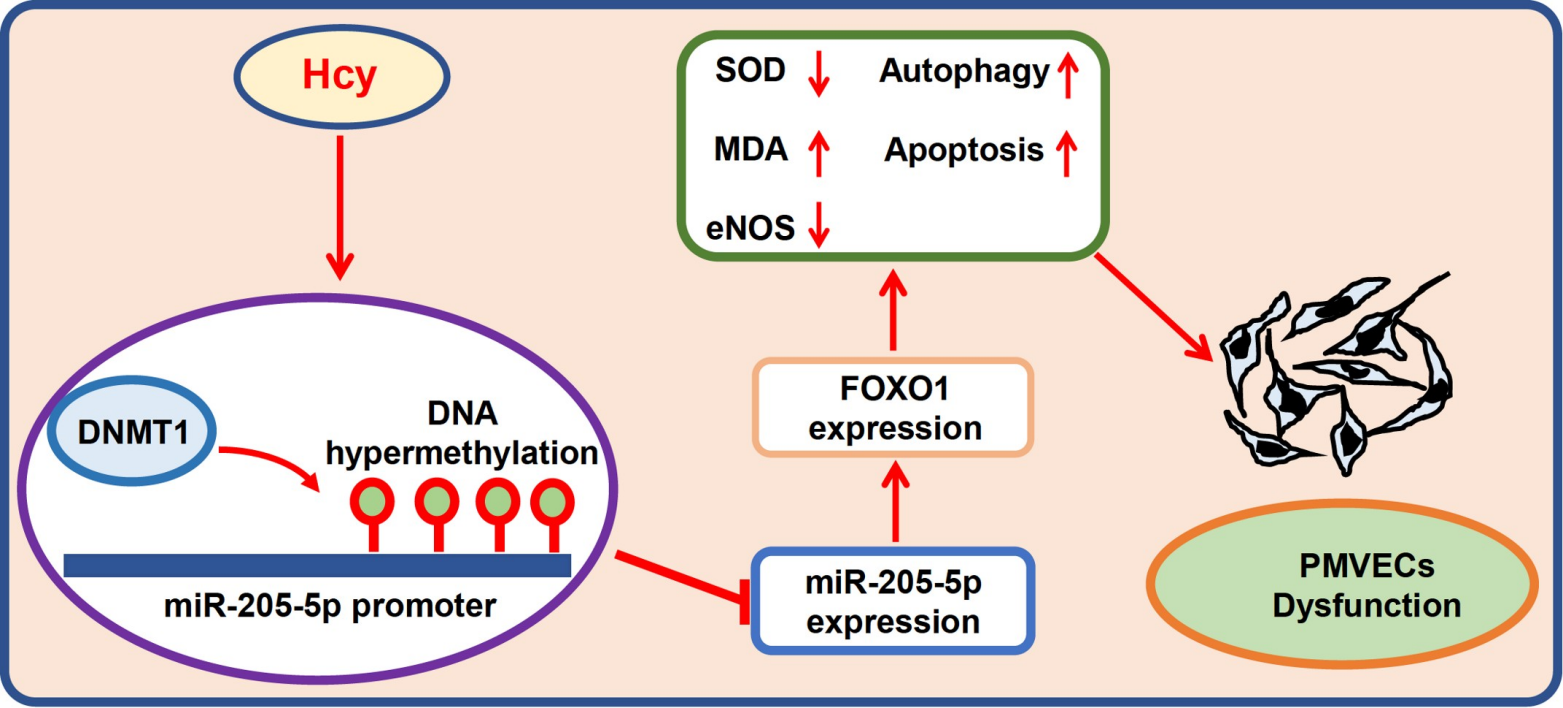
Figure 6 Proposed model of miR-205-5p expression regulation in Hcy-induced PMVEC dysfunction Under hyperhomocysteinaemia (HHcy), downregulation of miR-205-5p specifically upregulates FOXO1 expression in PMVECs. Then, FOXO1 induces pulmonary injury.

In summary, DNA hypermethylation inhibits miR-205-5p transcription in Hcy-induced PMVEC dysfunction. Downregulation of miR-205-5p accelerates PMVEC dysfunction by targeting FOXO1 ( Figure 6). Activation of miR-205-5p could be a potential therapeutic target to protect Hcy-induced lung microvascular function disorder.
